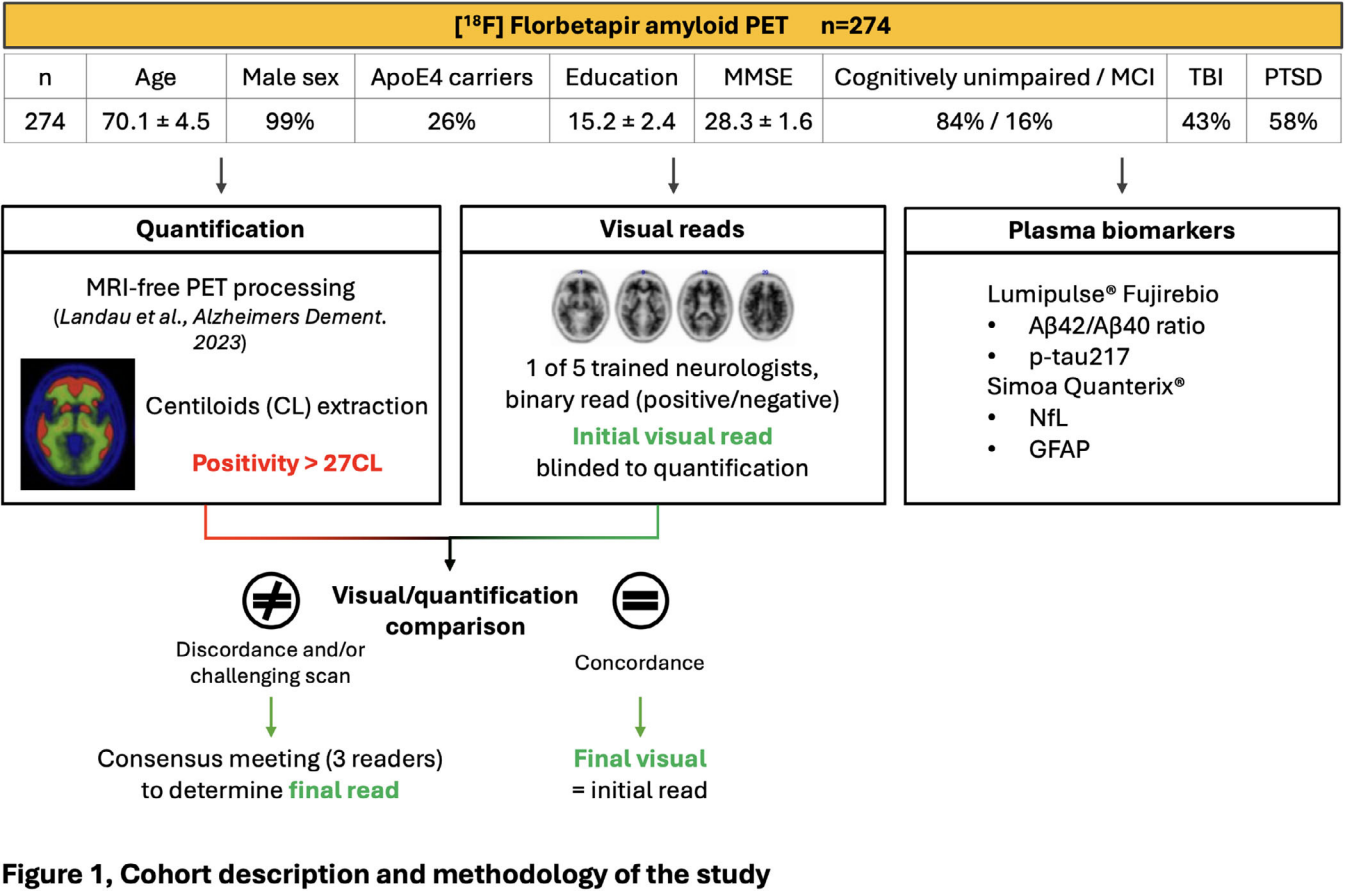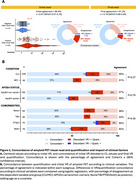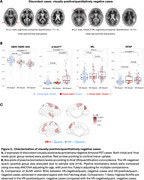# Evaluating agreement between visual read and quantification of amyloid PET in the ADNI‐DOD cohort

**DOI:** 10.1002/alz70856_100051

**Published:** 2025-12-25

**Authors:** Agathe Vrillon, Ganna Blazhenets, Yael Rosen Lang, Julien Lagarde, Konstantinos Chiotis, Maison Abu Raya, David N. Soleimani‐Meigooni, Gil D. Rabinovici, Michael W Weiner, Lisa C Silbert, Nathan Hantke, Daniel Schwartz, Abigail Livny, Orit H. Lesman‐Segev, Kristine Yaffe, Leslie M. Shaw, Susan M. Landau, Raquel C. Gardner, Renaud La Joie

**Affiliations:** ^1^ Global Brain Health Institute, San Francisco, CA, USA; ^2^ Memory and Aging center, UCSF, San Francisco, CA, USA; ^3^ Memory and Aging Center, Weill Institute for Neurosciences, University of California San Francisco, San Francisco, CA, USA; ^4^ Sheba Medical Center, Ramat Gan, Israel; ^5^ Université Paris Cité, Paris, France; ^6^ Neurology of Memory and Language Department, GHU Paris Psychiatrie & Neurosciences, Hôpital Sainte‐Anne, Paris, France; ^7^ Global Brain Health institute‐ UCSF, San Francisco, CA, USA; ^8^ UCSF, San Francisco, CA, USA; ^9^ San Francisco Veterans Administration Medical Center (SFVAMC), San Francisco, CA, CA, USA; ^10^ Portland Veterans Affairs Medical Center, Portland, OR, USA; ^11^ NIA‐Layton Oregon Alzheimer's Disease Research Center, Oregon Health & Science University, Portland, OR, USA; ^12^ Oregon Health and Science University, Portland, OR, USA; ^13^ VA Portland Health Care System, Portland, OR, USA; ^14^ NIA‐Layton Aging & Alzheimer's Disease Center, Portland, OR, USA; ^15^ The Joseph Sagol Neuroscience Center, Sheba Medical Center, Tel Hashomer, Israel; ^16^ Department of Diagnostic Imaging, Sheba Medical Center, Tel Hashomer, Israel; ^17^ The Joseph Sagol Neuroscience Center, Sheba Medical Center, Ramat Gan, Israel; ^18^ Global Brain Health Institute, Memory and Aging Center, University of California San Francisco, San Francisco, CA, USA; ^19^ University of California San Francisco / San Francisco VA Medical Center, San Francisco, CA, USA; ^20^ University of California, San Francisco, Weill Institute for Neurosciences, San Francisco, CA, USA; ^21^ NCIRE‐The Veterans Health Research Institute, San Francisco, CA, USA; ^22^ Perelman School of Medicine, University of Pennsylvania, Philadelphia, PA, USA; ^23^ Neuroscience Department, University of California, Berkeley, Berkeley, CA, USA; ^24^ Sheba Medical Center, Joseph Sagol Neuroscience Center, Ramat Gan, Israel; ^25^ San Francisco Veterans Affairs Health Care System, San Francisco, CA, USA; ^26^ Global Brain Health Institute, University of California, San Francisco, San Francisco, CA, USA; ^27^ Memory and Aging Center, Weill Institute for Neurosciences, University of California, San Francisco, San Francisco, CA, USA

## Abstract

**Background:**

Traumatic brain injury (TBI) and post‐traumatic stress disorder (PTSD), among other comorbidities, are common in military Veterans. These factors could affect visual interpretation of amyloid PET. We evaluated the agreement between PET visual read (VR) and Centiloid (CL) quantification in the ADNI‐DOD cohort enrolling US Vietnam war Veterans.

**Method:**

All ADNI‐DOD participants who underwent [^18^F]Florbetapir amyloid PET were included (Figure 1). VR were performed by one of five trained neurologists blinded to demographics, clinical information, and PET quantification. CL quantification used an MRI‐free pipeline (Landau *et al.*, positivity threshold: CL >27). Visually and quantitatively discordant and/or challenging scans were reviewed in consensus meetings to achieve a final read. Plasma Ab42/40 ratio and *p*‐tau217 levels were measured using Lumipulse® and plasma NfL and GFAP levels, using Simoa®, in all participants. Agreement was evaluated using percentage of agreement and Cohen's kappa (κ). Regional Florbetapir‐SUVR values were extracted in standard space using the Flechsig atlas to compare VR‐positive/quantitatively‐negative cases to VR‐negative/quantitatively‐negative cases.

**Result:**

We included 274 Veterans (mean age: 70.1 [4.5] years; 99% male; 84% cognitively unimpaired; 16% mild cognitive impairment; TBI: 47%; PTSD: 58%). Overall, 36% (*n* = 100/274) of scans were initially read as positive and 24% (*n* = 67/274) were quantitatively positive. Initial visual read and quantification were concordant for 86% of scans (κ=0.67 [95%CI 0.57‐0.76]), Figure 2a). Agreement between VR and quantification was not significantly impacted by cognitive stage, PTSD or TBI, or APOE4 carriership (Figure 2b). Review of discordant or challenging scans in a consensus meeting increased the concordance between VR and quantification to 91% (κ=0.78 [95%CI 0.69‐0.85]). Visual/quantitative discordance (9%) was principally constituted of VR‐positive/quantitatively‐negative scans (83% [*n* = 20/24 of overall disagreement using final read]). VR‐positive/quantitatively‐negative scans were characterized mostly by focal tracer uptake or posterior patterns not captured by CL quantification (Figure 3a). VR‐positive/quantitatively‐negative cases displayed higher plasma GFAP levels than VR‐negative/quantitatively negative (Figure 3b) and increased SUVR in ROI analysis across parietal, frontal and temporal cortices (Figure 3c).

**Conclusion:**

We observed a high agreement between visual read and quantification of amyloid PET, which was robust to clinical factors. Visual read could contribute to identifying early or atypical AD patterns not captured by Centiloids.